# Humans exercising in the heat: A review on sweat models and a comparison to recent experimental datasets

**DOI:** 10.1080/23328940.2025.2508534

**Published:** 2025-06-05

**Authors:** Robin de Korver, Boris R. M. Kingma, George Havenith, Kalev Kuklane, Glen P. Kenny, Robert D. Meade, Arjan J. H. Frijns

**Affiliations:** a Department of Mechanical Engineering, Eindhoven University of Technology, Eindhoven, The Netherlands; b Department of Human Performance, The Netherlands Organization for Applied Scientific Research (TNO), Soesterberg, The Netherlands; c Department of Human Nutrition, University of Copenhagen, Copenhagen, Denmark; d Environmental Ergonomics Research Centre, School of Design and Creative Arts, Loughborough University, Loughborough, UK; e Dutch Academy for Crisis Management and Fire Service (NACB), Netherlands Institute for Public Safety (NIPV), Zoetermeer, The Netherlands; f Chair of Biosystems Engineering, Institute of Forestry and Engineering, Estonian University of Life Sciences, Tartu, Estonia; g Human and Environmental Physiology Research Unit, School of Human Kinetics, University of Ottawa, Ottawa, ON, Canada; h Department of Epidemiology, T.H. Chan School of Public Health, Harvard University, Boston, MA, USA

**Keywords:** Sweating, exercise, water loss, insensible, body temperature regulation, models, theoretical

## Abstract

Sweating is a vital thermoregulatory mechanism in humans for maintaining thermal balance during exercise and exposure to hot environments. The development of models that predict sweat rate based on body temperature has been ongoing for over half a century. Here, we compared predicted water loss rates (WLR) from these models to actual observations collected during 780 participant-exposures in three independent laboratory-based experiments. In these experiments, male participants aged 19–50 years cycled or walked at various intensities (metabolic heat productions between 200 and 970 W), in air temperatures ranging from −40°C to 50°C, relative humidities (14% to 95%), and air velocities (<0.2 to 10 m/s), while wearing different clothing ensembles (thermal insulation 0.20 to 3.75 clo). The models’ performances were evaluated by the coefficient of determination (R^2^) and Root Mean Square Error (RMSE). Performance varied greatly with a maximum R^2^ value of 0.5 and RMSE values ranging from 10.4 to 4.9 g/min. Models with a lower sweat onset core temperature setpoint performed better and most models generally underestimated the water loss at higher WLR. Optimization of the core and skin temperature setpoints suggests preferred core temperature setpoints within a narrow range (36.2°C to 36.6°C). Even with optimized inputs, R^2^ values were around 0.5, meaning only 50% of the variance in observed WLR was explained by the models. Better model consideration of relations between body temperature and sweat rate, and the incorporation of non-thermal exercise-induced sweat promotion, may reduce model underpredictions at higher exercise intensities.

## Introduction

Sweating is a vital physiological process crucial in regulating body temperature and maintaining homeostasis during heat stress [1]. When the body heats up due to exercise or exposure to heat, sweat glands secrete fluid onto the skin’s surface [2]. As this fluid evaporates, it dissipates heat, which can help to reestablish a state of thermal balance in compensable conditions and prevent overheating [3]. Therefore, predicting sweat rate is important for assessing and managing heat stress, especially in environments where individuals are exposed to high temperatures for prolonged periods, and many models have been developed for this purpose [4–10]. By accurately forecasting sweat production, it becomes possible to design effective hydration strategies, monitor the risk of dehydration, and prevent heat-related illnesses such as heat stroke or heat exhaustion [11]. This is particularly critical in occupational settings (e.g. construction, military, or emergency response) and sports, where performance and safety can be compromised by inadequate thermal regulation [12].

Key research on the role of sweating in thermoregulation in humans emerged in the 1900s and continues today. In 1956, Kuno proposed that the regulation of sweating is mediated centrally [13], and was supported by work from Benzinger in 1959 that showed a relationship between sweat rate and body core temperature [14]. Later research demonstrated the modulating effects of peripheral (skin) temperature on the regulation of sweating [15–21]. Stolwijk, a pioneer in the field of thermophysiological modeling, used this core and skin temperature control principle in the development of the first multi-node thermophysiological model [5]. A thermophysiological model is a set of mathematical equations thought to describe the heat exchange of the human body with its environment and its physiological thermal response [10,22]. Thermophysiological models are used to assess the thermal state of the human body in different thermal conditions. This body temperature-based thermoregulation gained widespread adoption in many thermoregulation models, where sweating (and other thermoeffector responses such as shivering and vascular tone) are modeled as a function of the core and skin temperatures [4–10]. While the thermophysiological models that incorporate these sweat models are often validated – primarily by comparing their core and/or skin temperature predictions [9,10,23–26]—the sweat predictions of the sweat models have yet to be compared to independent empirical data.

In general, two different types of sweat models are prevalent in the literature. These are the core and skin temperature sweat models, which are investigated in the present paper, and the conceptual evaporative requirement ($E_{\mathrm{req}}$) sweat models. Both use a different methodology, the former is based on physiological principles of the human body (i.e. temperature signals), and the latter on thermodynamic principles (i.e. heat balance). $E_{\mathrm{req}}$ represents the evaporation energy required for thermal heat balance and is estimated via the thermal energy balance between metabolic heat production and heat exchange with the environment (excluding evaporation from sweat). Many versions exist. However, all are based on the principle that in response to elevations in environmental heat load and heat load generated by exercise, the human body produces and evaporates sweat to compensate for the excess heat required for heat balance (i.e. $E_{\mathrm{req}})$ [27–33]. Despite these models being widely employed, relatively simple, and showing promising results for group-averaged sweat predictions [32,33], changes in heat balance are not directly sensed by the body (and therefore could not regulate sweat rate via this pathway).

When the body gains heat, its temperature rises, activating deep-body core and peripheral temperature sensors that send signals to the brain (primarily the hypothalamus) [1,22,34], which in turn causes an increase in sudomotor activity (i.e. activating sweat glands) to facilitate heat dissipation through evaporation, helping to counteract further temperature rise. The core and skin temperature-controlled sweat models try to mimic this physiological process to predict the sweat rate. These models often consist of three components defined by core temperature, skin temperature, and local temperature effects (Q10). The core and skin temperature components are a function of the error signal. The error signal is defined by the difference between body temperature and the setpoint temperature (i.e. $T-T_{\mathrm{setpoint}}$). Note that the setpoint in this context is the onset temperature for sweat activation, equivalent to the thermoeffector threshold value defined by Romanovsky [35]. The magnitude of the core temperature function is often significantly larger than that of the skin temperature; indicating the larger influence of core temperature in the regulation of sweating. Additionally, local skin temperature affects the activation and production of sweat within the affected area, which is modeled by the Q10 effect. The Q10 effect refers to the rate of changes in chemical or physiological processes to a 10°C temperature change [36]. Various studies investigated the Q10 effect of the local skin temperature on local sweating in humans and often reported a Q10 coefficient between 2 and 3 [20,37,38].

In this study, we first overview advances in temperature-regulated sweat models and then compare them with independent experimental data. To achieve our objective, we assessed several commonly used temperature-regulated sweat models.

## Materials and methods

### Sweat models

We compare several commonly used temperature-regulated sweat models that include: the Stolwijk model [5], the Wissler model [10], the JOS models [8,9], the Fiala model [4,39], and the sweat models developed by Havenith [6,7]. For uniformity, all sweat rates are given in mg·cm^−2^·min^−1^. If necessary, the corresponding (original) formula for the sweat rate is adjusted accordingly.

The sweat models predict the sweat rate, Sw, based on body core and skin temperatures. While the exact formula varies from model to model, they generally conform to the following expression:

(1) $$\mathrm{Sw}=\;f\left(T_{c}-T_{c,\mathrm{set}}\right)+g\left({\bar{T}}_{\mathrm{sk}}-{\bar{T}}_{\mathrm{sk},\mathrm{set}}\right)\;\cdot Q_{10}$$

Equation 1 can be divided into three distinct components, 1) $f\left(T_{c}-T_{c,\mathrm{set}}\right)$; a function of the core temperature error signal with $T_{c,\mathrm{set}}$ the core temperature setpoint at which sweating begins, 2) $g\left({\overset{ˉ}{T}}_{\mathrm{sk}}-{\overset{ˉ}{T}}_{\mathrm{sk},\mathrm{set}}\right)$; a function of the mean skin temperature error signal with ${\overset{ˉ}{T}}_{\mathrm{sk},\mathrm{set}}$ the mean skin temperature setpoint for the onset of sweating, and 3) $Q_{10}$; a multiplicative factor that captures the metabolic influence of the local skin temperature on sweat gland activity.

The first sweating model that is considered is the sweating model developed by Stolwijk et al. [5] in the 1960s and 1970s. The sweat rate formula is given by Equation 2.

(2) $$\mathrm{Sw}=\left[\left(E_{\mathrm{basal}}+\left(320\cdot \left(T_{c}-36.96\right)\;\;\;\;\;\;\;\;\;\;\;\;\;\;\;\;+29\cdot \left({\overset{ˉ}{T}}_{\mathrm{sk}}-34.08\right)\right)\cdot 2^{\frac{{\overset{ˉ}{T}}_{\mathrm{sk}}-34.08}{10}}\right)\right]\cdot \frac{C_{\mathrm{kCal}/h}}{A_{\mathrm{du}}}$$

Here, Sw [mg·cm^−2^·min^−1^] is the sweat rate, $T_{c}$ [°C] is the core temperature, ${\overset{ˉ}{T}}_{\mathrm{sk}}$ [°C] is the mean skin temperature, and $E_{\mathrm{basal}}$ [kCal·h^−1^] is the basal evaporative heat loss of the skin, which represents water diffusion through the skin and has a value of 9.0 kCal·h^−1^ [5]. $C_{\mathrm{kCal}/h}$ ( = 28.70) is the conversion constant to convert the sweat rate from kCal·h^−1^ to mg·min^−1^ and $A_{\mathrm{du}}$ [cm^2^] is the body surface area of the participant based on Du Bois and Du Bois [40]. The mean skin temperature setpoint in this equation (34.08°C) is the weighted average setpoint of the skin temperature following the methodology described by Stolwijk [5]. The core temperature setpoint (36.96°C) represents the temperature at the hypothalamus as is also described by Stolwijk [5].

The JOS-2 model [8] is a thermophysiological model based on Stolwijk’s model. It uses a sweat rate formula that is similar to that of Stolwijk; however, it employs different temperature setpoint values and includes a factor to scale for the body surface area:

(3) $$\mathrm{Sw}=\left[\left(371.2\cdot \left(T_{c}-37.06\right)+33.64\;\;\;\;\;\;\;\;\;\;\;\;\cdot \left({\overset{ˉ}{T}}_{\mathrm{sk}}-34.53\right)\right)\cdot \frac{A_{\mathrm{du}}}{A_{\mathrm{du},\mathrm{st}}}\cdot 2^{\frac{{\overset{ˉ}{T}}_{\mathrm{sk}}-34.53}{10}}\right]\cdot \frac{C_{W}}{A_{\mathrm{du}}}$$

where $A_{\mathrm{du}}$ [cm^2^] is the body surface area of the participant based on Du Bois and Du Bois [40] and $A_{\mathrm{du},\mathrm{st}}$ [cm^2^] is the standard body surface area, for which they assume 1.87 m^2^ [8]. $C_{W}$ ( = 24.69) is the conversion constant to convert the sweat rate from W to mg·min^−1^. The given mean skin temperature setpoint (34.53°C) is the weighted average setpoint of the skin temperature following the methodology described by Kobayashi et al. [8] and the core temperature setpoint (37.06°C) represents the setpoint of the head core (i.e. hypothalamus) [8].

Next is the JOS-3 thermophysiological model [9], which defines sweating responses using the following equation:

(4) $$\mathrm{Sw}=\left[\left(371.2\cdot \left(T_{c}-37.46\right)+33.64\;\;\;\;\;\;\;\;\;\;\cdot \left({\overset{ˉ}{T}}_{\mathrm{sk}}-34.62\right)\right)\cdot \frac{A_{\mathrm{du}}}{A_{\mathrm{du},\mathrm{st}}}\cdot AG_{\mathrm{sweat}}\cdot 2^{\frac{{\overset{ˉ}{T}}_{\mathrm{sk}}-34.62}{10}}\right]\cdot \frac{C_{W}}{A_{\mathrm{du}}}$$

where $AG_{\mathrm{sweat}}$ [-] is the aging factor for sweat. This factor represents the decreased sweat rates observed in older adults, and its value is based on a study by Inoue et al. [41]. The weighted average value of $AG_{\mathrm{sweat}}$ is 0.53 for people older than 60 years and is one for people younger than 60 [9]. The primary changes with JOS-3 sweat model compared to JOS-2 include the addition of a decreased sweat rate for older adults and an increase in both the core and mean skin temperature setpoints to 37.46°C and 34.62°C respectively.

In addition, the thermophysiological model of Fiala [4,39] is based on the Stolwijk model and is defined by the following equation:

(5) $$\mathrm{Sw}=\left[\left(\left(0.8\cdot \tanh\left(0.59\cdot \left({\overset{ˉ}{T}}_{\mathrm{sk}}-34.4\right)\;\;\;\;-0.19\right)+1.2\right)\cdot \left({\overset{ˉ}{T}}_{\mathrm{sk}}-34.4\right)\;\;\;\;\;\;\;\;\;\;\;\;\;\;\;\;\;\;\;+\left(5.7\cdot \tanh\left(1.98\cdot \left(T_{c}-37\right)-1.03\right)+6.3\right)\cdot \left(T_{c}-37\right)\right)\cdot 2^{\frac{{\overset{ˉ}{T}}_{\mathrm{sk}}-34.4}{10}}\right]\cdot \frac{C_{g/min}}{A_{\mathrm{du}}}$$

where $C_{g/min}$ ( = 1000) is the conversion constant converting the sweat rate from g·min^−1^ to mg·min^−1^. Like Stolwijk, JOS-2, and JOS-3, setpoints are used for mean skin temperature (34.4°C) and core temperature (37°C) [39]. However, in Eq. 5 the effect of a change in temperature signal (i.e. $T-T_{\mathrm{setpoint}}$) is non-linear (when excluding the Q10 effect) as it is incorporated into a hyperbolic tangent function.

In the above models, fitness, acclimation, and activity levels are not taken into account. The sweat rate formula proposed by Havenith [6,7] incorporates the effects of the individual’s fitness (quantified by $\dot{V}O_{2\max}$ the maximum rate of oxygen consumption) and acclimation status on the sweat rate and is given by [7]:

(6) $$\mathrm{Sw}=\;g_{\mathrm{sw}}\cdot \left(0.92\cdot \mathrm{csw}\cdot \left(T_{c}-\left(37+T_{c,\mathrm{offset}}\right)\right)+0.08\cdot \mathrm{csw}\cdot \left({\bar{T}}_{\mathrm{sk}}-33.7\right)\right)\cdot \exp\left(\frac{\max\left\{0,{\bar{T}}_{\mathrm{sk}}-33.7\right\}}{10.7}\right)\;\cdot C_{g/m^{2}/h}$$

where $g_{\mathrm{sw}}$ is the gain factor given by Equation 7, csw (170 g·m^−2^·h^−1^) is a sweat rate constant, $T_{c,\mathrm{offset}}$ [°C] is given by Equation 10 and is the shift in the core temperature setpoint based on the individual’s fitness and acclimation status, and

$C_{g/m^{2}/h}$ ( = 1.67·10^−3^) is the conversion constant to convert the sweat rate from g·m^−2^·h^−1^ to mg·cm^−2^·min^−1^. The effect of fitness and acclimation status (i.e. being acclimated or not) is represented by a change in the sweating onset temperature ($T_{c,\mathrm{offset}}$) and slope ($g_{\mathrm{sw}}$). A higher fitness and/or being acclimated decreases the sweating onset temperature and increases the slope of the sweat rate. Havenith [7] also uses a slight variation of the traditional Q10 effect with the main difference being that this Q10 will always be equal to or higher than 1 (i.e. no sweat rate decrease at lower skin temperatures (as Q10 is capped at a minimum value of 1) only an increase at elevated skin temperatures).

The gain factor, $g_{\mathrm{sw}}$, affects the slope of the sweat rate based on the individual’s fitness and acclimation status, and is described as [6,7]:

(7) $$g_{\mathrm{sw}}=\left(1+0.35\cdot \frac{\mathrm{fit}}{20}\right)\cdot \left(1+0.15\cdot f_{\mathrm{ac}}\right)$$

where fit is the individual’s fitness level, which is based on the individual’s maximum aerobic capacity, see Equation 8, and $f_{\mathrm{ac}}$ is the individual’s acclimation status given by Equation 9 [6,7].

The individual’s fitness is given by [6,7]:

(8) $$\mathrm{fit}=\dot{V}O_{2\max}-\dot{V}O_{2\max,\mathrm{standard}}$$

where $\dot{V}O_{2\max}$ [ml·kg^−1^·min^−1^] is the individual’s maximum aerobic capacity with a range between 20 (unfit) and 60 (fit) ml·kg^−1^·min^−1^, and $\dot{V}O_{2\max,\mathrm{standard}}$ [ml·kg^−1^·min^−1^] is the maximum aerobic capacity of the average person, which is taken to be 40 [6,7].

The individual’s acclimation status $f_{\mathrm{ac}}$, with values ranging between 0 (not acclimated) and 1 (fully acclimated), depends on the number of acclimation days $n_{d}$. It is described as [6,7]:

(9) $$f_{\mathrm{ac}}=1-\exp\left(-0.3\cdot \left(n_{d}-1\right)\right)$$

where the number of acclimation days is ranging between 0 and 14 [6,7].

The individual’s core temperature setpoint offset is given by [6,7]:

(10) $$T_{c,\mathrm{offset}}=-\left(0.1\cdot \frac{\mathrm{fit}}{10}+0.25\cdot f_{\mathrm{ac}}\right)$$

The maximum sweat rate depends on acclimation status and fitness following:

(11) $$Sw_{\max}=Sw_{\max,\mathrm{standard}}\cdot \left(1+0.25\cdot \frac{\mathrm{fit}}{20}+0.25\cdot f_{\mathrm{ac}}\right)$$

where $Sw_{\max,\mathrm{standard}}$ (800 g·m^−2^·h^−1^ [7]) is the maximum sweat rate for an unacclimated individual with a $\dot{V}O_{2\max}$ of 40 ml·kg^−1^·min^−1^.

Wissler proposes three sweat rate equations based on the individual’s acclimation status and whether the individual is resting or exercising [10]. Eq. 12 is for rest and unacclimated, Eq. 13 is for exercise and unacclimated or rest and acclimated, and Eq. 14 is for exercise and acclimated [10].

(12) $$\mathrm{Sw}=0.327\cdot \left(T_{c}+0.15\cdot {\overset{ˉ}{T}}_{\mathrm{sk}}-42.1\right)$$

(13) $$\mathrm{Sw}=0.327\cdot \left(T_{c}+0.15\cdot {\overset{ˉ}{T}}_{\mathrm{sk}}-41.6\right)$$

(14) $$\mathrm{Sw}=0.327\cdot \left(T_{c}+0.15\cdot {\overset{ˉ}{T}}_{\mathrm{sk}}-41.1\right)$$

Through these equations, Wissler simulates the effects of exercise and acclimation as defined by a decrease in the sweating onset temperature. Notably, this approach differs from Havenith, where acclimation status is defined by both a reduction in the onset for sweating and an increase in slope (thermosensitivity of the sweat response). While Wissler’s equations consider skin temperature, they neglect the Q10 effect [10].

**Figure 1.**
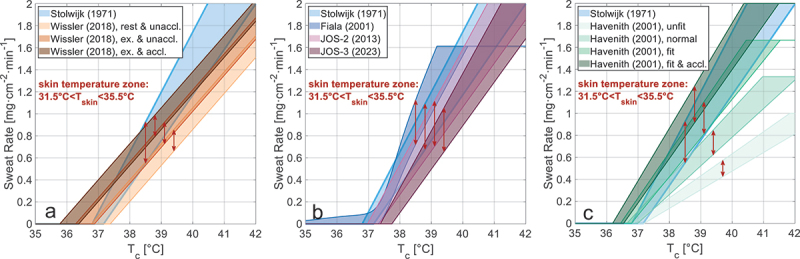
Predicted sweat rates as a function of the core temperature and a range of comfortable skin temperatures (i.e. 31.5°C to 35.5°C [42]), shown in three parts: (a) Stolwijk and Wissler models, (b) Fiala, Stolwijk, JOS-2, and JOS-3 models, and (c) Stolwijk and Havenith models.

Figure 1 shows the predicted sweat rates of the models for an increasing core temperature and assuming skin temperatures between 31.5°C and 35.5°C (double-headed red arrows), which corresponds to a comfortable thermal condition [42]. To help compare all models, Stolwijk (arbitrarily chosen) is given in all three subfigures with enlarged boundary lines. The models overviewed in these figures highlight that the sweat rate of all models increases with rising core and skin temperatures. However, it also illustrates significant variations in the predicted sweat rates among different models. For example, at T_c_ = 39°C, the Fiala sweat model (dark blue area) predicts a sweat rate of ~1.24 mg·cm^−2^·min^−1^, which is double that predicted by the Havenith normal model (green area), ~0.62 mg·cm^−2^·min^−1^.

### Experiments

For the development of most sweat models (Stolwijk, Fiala, and Wissler), the NASA dataset [43] was used. In the present study, three different independent datasets were used to compare the described sweating formulae. The first dataset is from the University of Ottawa (uOttawa) [44], which investigated the effect of the required evaporation for heat balance and relative exercise intensity (%$\dot{V}O_{2\max}$ on sweat rate. The second dataset contains two EU-funded projects, which are the Subzero (SZ) and Thermprotect project (TP) [45,46]. The SZ project assessed the reliability and accuracy of thermal manikins for cold protective clothing [45] and the TP project examined the thermal strain when wearing protective clothing [46]. The last dataset is also a European Union (EU)-funded project called HEAT-SHIELD, which aimed to address the negative impacts of heat stress experienced by the EU workforce [47–50].

Important to note is that in all three datasets, whole-body sweat loss (WBSL) or whole-body sweat rate (WBSR) was not measured directly. Instead, total water (or weight) losses were measured, which consist mostly of the water losses from generated sweat but also contain the water losses from the respiratory tract (added moisture in exhaled air).

The number of participants in each dataset and the characteristics (trial-weighted average and standard deviation) of the participants are shown in Table 1. Each dataset is described in detail in the following sections.

**Table 1.** Trial-weighted participant characteristics (mean ± SD).

Characteristics	uOttawa [44]	Subzero + Thermprotect [45,46]	HEAT-SHIELD [47–50]	Combined
Gender	25M	32M	33M	90M
Age [y]	29.8 ± 10.5	26.9 ± 5.2	24.1 ± 2.8	25.5 ± 5.3
Height [m]	1.81 ± 0.07	1.79 ± 0.05	1.78 ± 0.06	1.79 ± 0.06
Mass [kg]	82.8 ± 8.2	77.3 ±10.3	75.6 ± 10.6	76.9 ± 10.5
Surface area [m^2^]^1a^	2.03 ± 0.12	1.95 ± 0.13	1.93 ± 0.15	1.94 ± 0.14
$\dot{V}O_{2\max}$ [mL·kg^−1^·min^−1^]	51.1 ± 6.7^b^	–	51.8 ± 8.4	–

^a^Method by Du Bois and Du Bois [40].

^b^Participant averaged (trial averaged data not available).

#### Dataset uOttawa

Dataset uOttawa consists of a study where water loss rate (WLR) data were collected to examine the independent contributions of the required evaporation for heat balance and of %$\dot{V}O_{2\max}$ to non-steady-state and steady-state WLR, irrespective of exercise intensity and ambient temperature [44]. In this study, 25 healthy males clothed in shorts and sandals cycled for 90 min in the modified Snellen air calorimeter (a device that provides a precise measurement of the heat dissipated by the human body [51]). Participants cycled at a fixed rate of heat production of 200, 350, and 500 W in a warm environment (ambient temperature of 30°C and absolute humidity of ~1.3 kPa). Trials were also conducted at a fixed rate of heat production of 290 W at increasing levels of ambient heat stress (i.e. ambient temperature of 30°C, 35°C, 40°C, and 45°C and absolute humidity of ~0.8 kPa). In total 94 experiments were performed; however, due to incomplete sensor data in some of the experiments, only 83 runs are used in the present paper.

Rectal temperature was measured with a thermocouple temperature probe (Mallinckrodt Medical Inc., St Louis, MO, USA) inserted 12 cm past the anal sphincter. Skin temperatures were measured with thermocouples (Concept Engineering, Old Saybrook, CT, USA) at four sites (upper trapezium, chest, quadriceps, and back calf). Rectal and skin temperatures were sampled every 15 s. Mean skin temperature was estimated using weights proposed by Ramanathan [52]. Time series data of the WLR were obtained from air calorimeter measurements. This modified Snellen air calorimeter is a cylindrical device (with a height of 1.83 m and a diameter of 1.68 m) that controls and measures the temperature and absolute humidity of the air entering and leaving the chamber [53]. Thereby, this device provides a precise measure of evaporative and dry heat exchange. It measures the air temperature with high precision thermistors (Black Stack model 1560, Hart Electronics, Fluke Corp., American Fork, UT, USA) and the absolute humidity with high precision dew point hygrometers (RH Systems model 373 h, Albuquerque, NM, USA). Both measurements were collected every 8 s. To account for all evaporation and heat losses, the exhaled air was recycled back into the calorimeter. The airflow through the calorimeter was estimated through differential thermometry of a known regulated heat source. Metabolic rate was estimated using electrochemical gas analyzers (AMETEK model S-3A/1 and CD 3A, Applied Electrochemistry, Pittsburgh, PA, USA) that measured the exhaled air oxygen and carbon dioxide concentrations. Metabolic heat production was calculated as metabolic rate minus the rate of external work. The external work on the ergometer was measured with a resistance control unit located outside the calorimeter.

#### Dataset SZ+TP

Dataset SZ+TP consists of two EU projects, the Subzero (SZ) [45] and Thermprotect (TP) project [46]. In total, 224 trials were performed, none of which had missing sensor data, and therefore are included in the dataset. The Subzero project aimed to assess the accuracy and reliability of thermal insulation measurements for cold protective clothing using thermal manikins, and to understand the factors influencing these measurements [45]. Four test institutes each performed 64 trials with 8 human participants. In the present paper, only the trials from two of the four test institutes (P5 and P6) are used as these institutes tested conditions that allowed higher heat storage in the test persons by using higher clothing insulation values and metabolic rates, leading to higher water losses. In these 128 trials, participants were clothed in one of three cold protective clothing ensembles and walked on a treadmill for 90 minutes. Each trial was conducted at a constant speed, air velocity, and air temperature. However, these conditions varied between different trials, with walking speeds ranging from 3.5 to 5 km/h, air temperatures from −40 to −10°C, and air velocities from 0.3 to 10 m/s. Additionally, trials were conducted both with and without a Sympatex membrane (water vapor barrier) in the outer layer.

Rectal temperature was measured with a temperature probe (YSI 700 or YSI 401, Yellow Springs Instrument, USA) inserted 10 cm beyond the anal sphincter. Skin temperatures were measured (YSI 400 or YSI 409b, Yellow Springs Instrument, USA) at 8 sites: forehead, right scapula, left upper chest, right upper arm, right forearm, right dorsal hand, right anterior thigh, and right calf. Mean skin temperature was estimated using the weights described in ISO 9886:2004 [54]. Air temperature was measured with thermistors. All temperatures were sampled once per minute. Water loss was determined by weighing the participants before and after exercise using a scale (Mettler ID1 MultiRange, Albstadt, Germany, 2 g resolution, and ±5 g accuracy). Metabolic rate was determined from oxygen consumption (Medikro 919 Ergospirometer, Finland or SensorMedics Corporation, Ca., USA) measured for the last 5 min of exercise. The average value of the last 2 min was used as the representative value.

The Thermprotect project (TP) investigated thermal strain experienced in personal protective clothing focusing on the effects of radiation, wind, and wetting [46]. The project involved modeling, material tests, thermal manikin tests, and human participant tests. In this study, the experimental data from human participant tests for work packages 2 and 3 (WP2 and WP3) are used.

The WP3 experiments were complementary to the Subzero trials. In this experiment, eight participants performed 4 trials each in which they walked on a treadmill for 90 min at a constant speed, air velocity, and air temperature. Walking speed (3 to 4.9 km/h) and air temperature (−25 or −10°C) were varied between trails. Trials were performed with and without a Sympatex membrane. Participants wore one of four ensembles of which two (garments B and C) were the same as used in the Subzero project and two were modified versions.

The WP2 experiments [55] from the Thermoprotect project focused on the effect of moisture in protective clothing on heat transfer. Eight participants performed 8 trials each in which they walked on a treadmill at 4.5 km/h for 60 min starting with wet or dry underwear (cotton shirt and trousers) with a permeable or impermeable coverall at an air temperature of 10°C or 25°C.

In both WP2 and WP3, rectal temperature was measured with a temperature probe (YSI-401 Yellow Springs Instrument, USA) inserted 10 cm beyond the anal sphincter. Skin temperatures were measured using NTC thermistors (ACC-001, Rhopoint Components Ltd, UK) at eight sites: forehead, right scapula, left upper chest, right upper arm, right forearm, right dorsal hand, right anterior thigh, and right calf. Mean skin temperature was estimated using the weights described in ISO 9886:2004 [54]. All temperatures, including air temperature (PT100, 1/10 Class B sensor), were sampled every 15 s. Water loss was determined by weighing the participants before and after exercise using a scale (KC 240 GWB Mettler ID2 MultiRange, Germany, 2 g resolution and ±5 g accuracy). Metabolic rate was determined via oxygen consumption measurements taken every 30 min in WP3 and between the 10^th^ and 20^th^ min in WP2.

#### Dataset HEAT-SHIELD

HEAT-SHIELD was an EU-funded project that addressed the negative impacts of heat stress experienced by EU workers at their workplaces. This dataset consists of experiments investigating the effects of temperature, humidity, wind speed, solar radiation, and clothing on physical work capacity [47–50,56].

Before the experimental trials, participants performed a submaximal exercise test on a graded treadmill to obtain their $\dot{V}O_{2\max}$ (Quark CPET, COSMED, Albano Laziale, Rome) [47,50]. In the experimental trials, participants (young healthy males) walked on a treadmill for up to 1 h, with the treadmill automatically adjusting its speed and incline to maintain a target heart rate of 130 beats per minute (bpm). The treadmill initially set the walking speed to 6 km/h and adjusted the incline to keep the heart rate at 130 bpm (i.e. a fixed cardiovascular strain protocol). If the heart rate continued to rise above 130 bpm despite the incline being set to zero, the treadmill reduced the walking speed. If the heart rate remained above 130 bpm even after the speed was reduced to zero, the trial was terminated. The trial was also terminated if the rectal temperature of the participants rose above 39°C. Trials were performed at different ambient air temperatures (ranging from 15°C to 50°C), humidities (20% to 80%), air velocities (<0.2 to 3.5 m·s^−1^), solar radiations (0 and 800 W·m^−2^), and two different sets of clothing (with intrinsic clothing insulation of 0.04 and 0.133 m^2^·K·W^−1^ (0.26 or 0.86 Clo) and evaporative resistance of 0.007 and 0.024 m^2^·Pa·W^−1^) [47–50,56]. In the present paper, only the trials with available and complete time series data are used, comprising data from 473 trials (out of the 816) from 33 participants.

Rectal temperature was measured with a rectal thermistor (VIAMED, Yorkshire, UK) 10 cm past the anal sphincter. Skin temperatures were measured using skin thermistors (Grant Instruments Ltd, Corby, UK) at four sites (chest, tricep, thigh, and calf) with mean skin temperature estimated via the proposed weights by Ramanathan [52], except for experiments with solar radiation. Here, skin temperatures were measured at six sites (upper back, lower back, chest, triceps, quadriceps, and calf) and the Ramanathan equation was adapted to include the effect of solar radiation [49]. For ambient conditions, wet-bulb globe temperature (WBGT; Quest temp model 34), air temperature, relative humidity, and air velocity (Testo Ltd, model 435–2 Alton, Hampshire, UK) were measured. These measurements (including core and skin temperatures) were sampled every minute. Water loss was determined by weighing the participants before and after exercise using a scale (Mettler Toledo KCC150, Leicester, UK, 1 g resolution and ±10 g accuracy). Metabolic rate was determined via the method proposed by Ludlow and Weyand [57]. This method uses the walking velocity and grade to estimate the metabolic rate for walking on the treadmill.

The experimental conditions for all datasets are summarized in Table 2. It is important to note that for all datasets, the number of trials exceeds the number of participants. Each participant completed multiple trials, ranging from 2 to 59 with a median of 8; however, no participant ever repeated the same condition. While this may reduce the overall variability (and variance), a total of 90 participants, of which 78% completed 10 or fewer trials, still ensures a wide range of individual responses.

**Table 2.** Dataset characteristics.

Characteristics	uOttawa [44]	Subzero (SZ) + Thermprotect (TP) [45,46]	HEAT-SHIELD [47–50]
Trials	83	224	473
Exercise period [min]	60	60 or 90	Max 55
Activity	Ergometer	Treadmill	Treadmill
Intensity control method	Fixed metabolic rate	Fixed walking speed	Fixed heart rate (130 bpm; variable speed and grade)
Country	Canada	Finland, Sweden, and Norway	UK
Clothing	Cotton underwear, shorts, socks, and athletic shoes	Cotton underwear (including socks, shirt, and trousers), and shoes or (heavy) winter clothing consisting of underwear an intermediate layer, handwear, and sometimes an outer garment and headgear	Either low clothing: underwear, standardized shorts, socks and trainers, or high clothing: low clothing + standardized cotton t-shirt and full body protective overall (65% polyester, 35% cotton)
Clothing insulation (static) [clo]	≈0.2–0.3	SZ: 2.93 to 4.13 TP: 1.55 to 3.81	0.26 and 0.86
Clothing water vapor resistance (static) [m^2^·kPa·W^−1^]	-	SZ: 0.033 to 0.11 TP: 0.020 to 0.14	0.007 to 0.024
Mean skin temperature calculation method	Ramanathan (upper trapezius, chest, quadriceps, calf	8-point ISO9886 (forehead, scapula, chest, upper arm, lower arm, hand, anterior thigh, calf)	Ramanathan (pectoralis major, triceps, rectus femoris, gastrocnemius)
Ambient temperature [°C]	30, 35, 40, 45	−40, −25, −10, 10, 25	15, 25, 30, 35, 40, 45, 50
Relative humidity [%]	14 to 31	32 to 95	20 to 80 (20, 50, and 80 most common)
Air velocity [m·s^−1^]	≲0.3	SZ: <0.2 to 10 TP: 0.3	<0.2 or 3.5
Metabolic heat production [W]	200, 290, 350, 500 (on average ~350)	210 to 850 (on average ~400)	220 to 970 (on average ~500)

### Evaluation

As stated before, the three datasets did not directly measure the sweat rate. Instead, we look at the water loss rate (WLR) which is defined as the water loss (WL) over the experimental time interval. In datasets SZ+TP and HEAT-SHIELD, the WL is calculated from the difference in pre- and post-exercise body mass. In the uOttawa dataset, the WL is obtained by the summation of the evaporation measurements from the modified Snellen air calorimeter. The WL measurement ultimately consists of all weight losses during the exercise period, which can be represented as:

(15) $$\mathrm{WL}=m_{\mathrm{sw}}+m_{\mathrm{resp}}+m_{\mathrm{others}}$$

where $m_{\mathrm{sw}}$ [g] is the mass of excreted sweat (i.e. the WBSL), $m_{\mathrm{resp}}$ [g] is the mass of the water losses from the respiratory tract, and $m_{\mathrm{others}}$ [g] is the mass of any food or fluid intake and excretion. Notably, $m_{\mathrm{others}}$ is zero in all three datasets, as drinking and washroom breaks were not permitted during the exercise period.

To enable comparison between the predictions from the sweating models and the experimentally measured WL, we calculate the predicted WL based on model outputs. The sweat loss component, $m_{\mathrm{sw}}$, is obtained from the sweating models. The respiratory water loss component, $m_{\mathrm{resp}}$, is estimated following the method described in ISO 7933 [58], which involves calculating the evaporative heat loss from respiration, $E_{\mathrm{resp}}$ [W/m^2^] (see Equation 16), and subsequently converting it into mass loss, $m_{\mathrm{resp}}$ (see Equation 17). Since no fluid or food intake or excretion occurred during the trials (i.e. $m_{\mathrm{others}}$ is zero), the predicted WL is the summation of $m_{\mathrm{sw}}$ and $m_{\mathrm{resp}}$.

ISO 7933 states that the evaporation losses in the respiratory tract, $E_{\mathrm{resp}}$ [W/m^2^], can be calculated using [58]:

(16) $$E_{\mathrm{resp}}=0.00127\cdot M\cdot \left(59.34+0.53\cdot T_{\mathrm{amb}}-11.63\cdot P_{\mathrm{amb}}\right)$$

where M [W/m^2^] is the metabolic rate, $T_{\mathrm{amb}}$ [°C] is the ambient air temperature, and $P_{\mathrm{amb}}$ [kPa] is the ambient water vapor pressure. Next, $E_{\mathrm{resp}}$ is converted to grams of evaporated water in the respiratory tract, $m_{\mathrm{resp}}$, using:

(17) $$m_{\mathrm{resp}}=\frac{E_{\mathrm{resp}}\cdot A_{\mathrm{du}}\cdot \Delta t_{\exp}}{\Delta H_{\mathrm{evap}}}$$

where $A_{\mathrm{du}}$ [m^2^] is the body surface area of the participant based on Du Bois and Du Bois [40], $\Delta t_{\exp}$ [min] is the exercise period, and $\Delta H_{\mathrm{evap}}$ [J/g] is the heat of vaporization of water (which is 2430 J/g at 30°C [22]).

Since the exercise period varies with each protocol, we introduce the average water loss rate (WLR), defined as:

(18) $$\mathrm{WLR}=\frac{\mathrm{WL}}{\Delta t_{\exp}}$$

The quality of the WLR predictions is evaluated via both the coefficient of determination (R^2^, see Equation 19) and the root mean square error (RMSE, see Equation 20).

The coefficient of determination is calculated as [59,60]:

(19) $$R^{2}=1-\frac{\sum_{i=1}^{n}{\left({\hat{Y}}_{i}-Y_{i}\right)}^{2}}{\sum_{i=1}^{n}{\left({\overset{ˉ}{Y}}_{i}-Y_{i}\right)}^{2}}$$

where n is the number of data points, $\hat{Y}$ represents the model’s prediction, Y is the observation (experimental data), and $\overset{ˉ}{Y}$ is the mean of the observations. The numerator is the residual sum of squares (RSS), which reflects the model’s error, while the denominator is the total sum of squares (TSS), related to the variance of the data. As the RSS decreases (i.e. predictions more closely match observations), R^2^ increases toward a maximum value of 1. Conversely, an increase in RSS causes R^2^ to decrease, potentially even approaching -∞. Negative R^2^ values are rare in practice because, when a model is evaluated on the same dataset it was trained on, R^2^ cannot drop below 0 (for unbounded regression). However, since the sweat models in this study are evaluated on a separate and independent dataset from the one they were trained on, R^2^ can be negative. For further details and an illustrative case study, refer to Chicco et al. [60].

The root mean square error, RMSE, is calculated as:

(20) $$\mathrm{RMSE}=\sqrt{\frac{1}{n}\overset{n}{\underset{i=1}{\sum }}{\left({\hat{Y}}_{i}-Y_{i}\right)}^{2}}$$

A lower RMSE indicates a better fit of the model to the data, as it means the predicted values are closer to the actual values.

### Dataset characteristics

**Figure 2.**
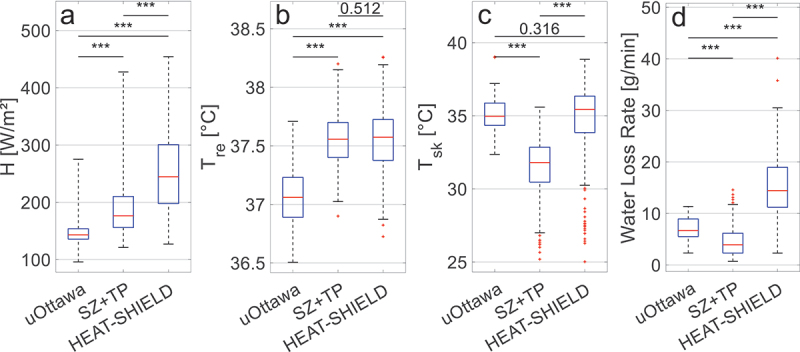
Boxplots of mean a) metabolic heat production, b) rectal temperature, c) skin temperature, and d) WLR of the datasets with significance levels (*: *p* < 0.05; **: *p* < 0.01; ***: *p* < 0.001).

Since the protocols of the experiments differ in terms of exercise intensity and environmental conditions, the resulting temperature distribution in the body can also vary. Therefore, we first examined whether metabolic heat production, core and skin temperatures, and WLR were significantly different (Figure 2).

From Figure 2, it is seen that the differences are all significant (*p* < 0.001) except for the rectal temperature between the SZ+TP and HEAT-SHIELD dataset (*p* = 0.512) and the skin temperature between the uOttawa and HEAT-SHIELD dataset (*p* = 0.316). Therefore, showing that the combined dataset consists of three distinctly different datasets; the uOttawa dataset has the lowest metabolic heat production and rectal temperature, high skin temperature, and medium WLR; the SZ+TP dataset has medium metabolic heat production, high rectal temperature, lowest skin temperature, and WLR; the HEAT-SHIELD dataset has the highest metabolic heat production and WLR, and both high rectal and skin temperatures. Note that these are the means of all trials of each dataset, individual trials can overlap.

## Results and discussion

**Figure 3.**
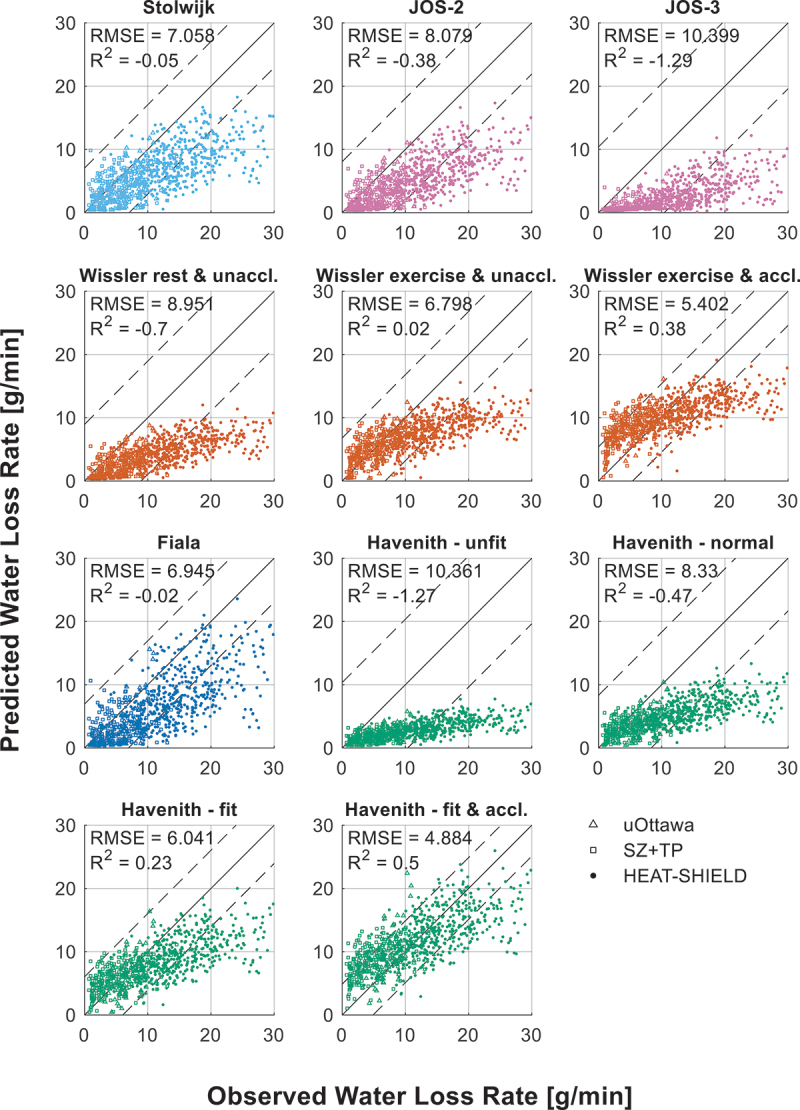
Predicted vs. observed water loss rate (WLR).

**Figure 4.**
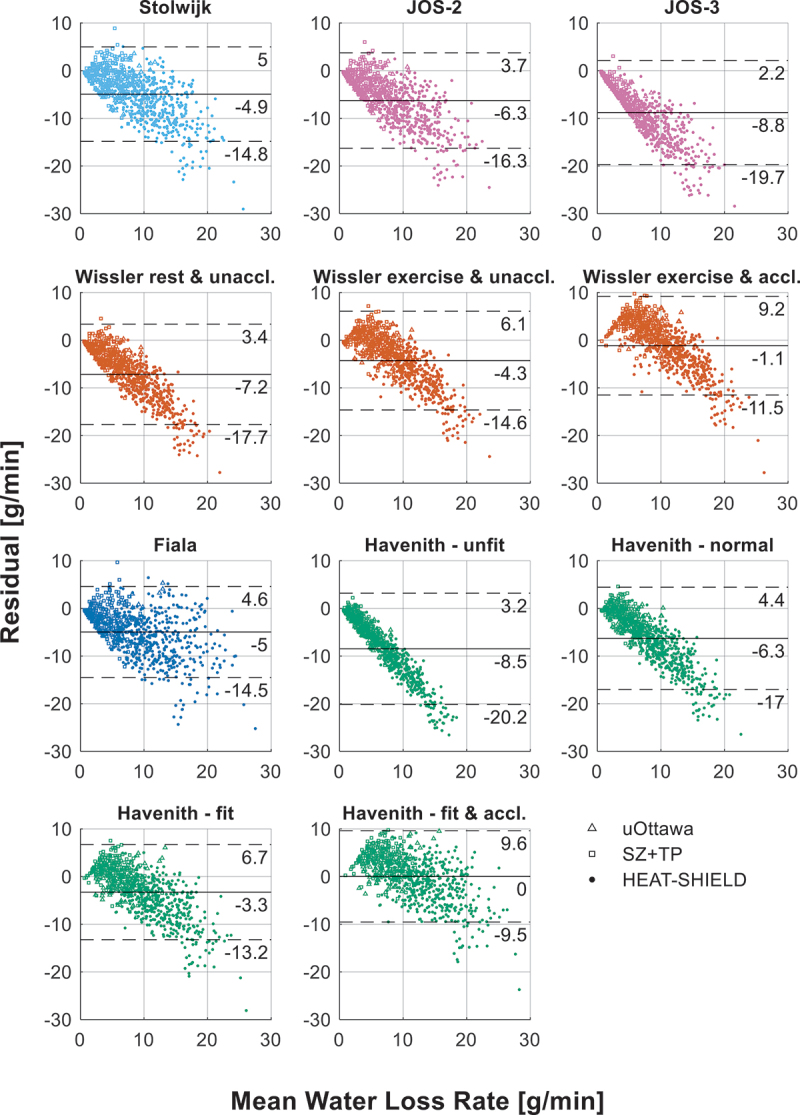
Bland-Atlman plot of the residual water loss rate (WLR), with the mean bias (black solid line) and limits of agreement (black dashed lines), and their corresponding values shown below each line.

Figure 3 shows the predicted and observed water loss rates. Each model is depicted in an individual subplot with their R^2^ and RMSE values and uniform axes for clarity and to prevent overlap. The two dashed black lines visualize the size of the RMSE of each model. The R^2^ of the models ranges between −1.3 and 0.5 and the RMSE between 4.9 and 10.4 g/min. Figure 4 shows Bland-Altman plots of the models with on the x-axis the mean of the predicted and observed WLR and on the y-axis the WLR difference (i.e. prediction minus observation). The solid line is the mean bias and the two dashed lines are the 95% limits of agreement (LoA). This figure demonstrates a clear trend in the prediction errors, which is that as the WLR increases the underpredictions of the models also increase. At low WLRs most models have a slight tendency to overestimate the WLR; however, this decreases as the WLR increases. This behavior is also captured in the mean bias, which is negative for all models except the Havenith fit and acclimated model and only slightly negative for the Wissler exercise and acclimated model.

Taken together, these findings suggest that while most models provide reasonable estimates at lower WLRs (approximately ≤10 g/min), their accuracy diminishes as WLRs increase. Given the systematic underprediction at higher values, caution is warranted when applying these models in high-heat-stress conditions. The extent to which these errors impact broader thermophysiological predictions remains unclear, as this would require further investigation into how inaccuracies in the estimation of WLR affect core and skin temperature predictions.

It is interesting to note that the majority of the models’ underpredictions are associated with the HEAT-SHIELD trials. When excluding the HEAT-SHIELD data, the acclimatized (and fit) models generally overpredict the WLR and are outperformed by their unacclimatized variants. One might conclude that the participants in the HEAT-SHIELD experiments were acclimatized. However, this is unlikely as precautions were taken to prevent acclimatization before and during the trials [47–50]. The reason for these underpredictions is discussed later in this study.

As is shown in Figure 1, the sweat-onset temperatures for the Havenith [6,7] and Wissler [10] models are lower in the acclimatized condition than in the non-acclimatized condition, suggesting that onset temperature might be a crucial parameter. To investigate this further, the core and skin temperature setpoints of all applicable sweat models were individually optimized using the dataset, by varying either the core or the skin temperature setpoint to maximize the R^2^ value. The Wissler model was not included in this optimization analysis, as it uses a single effective body temperature setpoint (a combination of core and skin temperatures) and does not permit independent adjustment of core and skin temperature thresholds. Additionally, the JOS-2 model is not optimized because of its similarity to the JOS-3 model. Among the Havenith sweat model variants, the Havenith fit model is selected as this best reflects the fitness ($\dot{V}O_{2\max}$ of the participants in the dataset. The results of these optimizations are shown in Table 3.

**Table 3.** Sweat models: core and skin temperature setpoint optimization results.

	Optimized core temperature setpoint			Optimized skin temperature setpoint		
Model	T_c,set,opt_	R^2^	RMSE [g/min]	$\overset{ˉ}{T}$_sk,set,opt_	R^2^	RMSE [g/min]
Fiala	36.59	0.43	5.21	32.38	0.47	5.00
JOS-3	36.32	0.52	4.78	27.53	0.45	5.10
Stolwijk	36.40	0.5	4.86	30.74	0.49	4.93
Havenith - Fit	36.15	0.49	4.93	31.28	0.53	4.69

Table 3 shows the optimized setpoint temperature and the model’s performance on the dataset with that optimized setpoint (i.e. R^2^ and RMSE). From this table, it is seen that all optimized setpoint temperatures are below the setpoints used in the original models, this is due to the original models on average underpredicting the WLRs (see Figure 4). The optimized core temperature setpoint fluctuates within a narrow range between 36.2°C and 36.6°C. No clear pattern or preferred temperature between the models is seen for the skin temperature setpoint. The R^2^ values of all models are relatively similar, fluctuating between 0.43 and 0.53, and the RMSE between 4.7 and 5.2 g/min.

**Figure A1.**
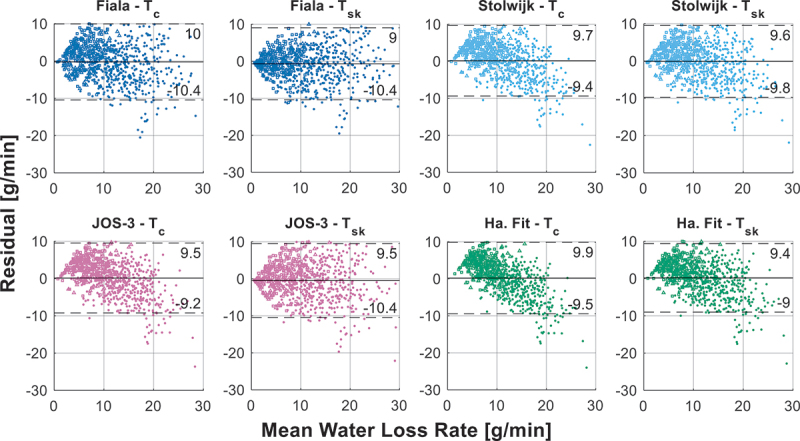
Bland-Altman plot of the residual water loss rate (WLR) of the models with either optimized core or skin temperature setpoint.

The Bland-Altman plot of the optimized models (Figure A1, Appendix – A) provides further insight into the linear error trend observed in Figure 4. Most models follow a linear function of the core and skin temperature error signals, except for the Fiala model, which follows an exponential function. This seems to suggest that the linear error trend in Figure 4 could be primarily due to an incorrect slope – specifically, a slope coefficient that is too low, as indicated by the predominant underpredictions. However, Figure A1 shows that adjusting the core or skin temperature setpoint could mitigate or even eliminate this trend without modifying the slope. This underscores the complexity of modeling sweat production and highlights the crucial role of setpoints in these models.

The uOttawa and most HEAT-SHIELD experiments used only four sites and weightings proposed by Ramanathan [52] to determine mean skin temperature. This method accounts for the majority of the data (approximately 500 out of 780 data points). The accuracy of determining mean skin temperature generally rises as the number of measured skin sites increases. As the skin temperature is an input for the sweat models, an error could arise from the low number of measured skin sites to estimate mean skin temperature. Nevertheless, Mitchell and Wyndham [61] performed experiments on nude men with a wide range of environmental conditions and compared several methods for calculating the mean skin temperature. The method of Ramanathan was one of the best scoring methods with an overall agreement frequency of 64% within 0.2°C and 98% within 1°C [61]. A more recent study by Liu et al. [62] reported similar results, with a 60% agreement frequency for experiments conducted in light clothing. Therefore, the estimated errors in measuring the mean skin temperature are below 1°C, which is acceptable. However, regardless of our definition of mean skin temperature, it is important to recognize that mean skin temperature serves only as a proxy for the neurological signals driving sweat gland activity. While this proxy is practical, the complex relationship between thermal state and sweat output is not fully understood.

Another source of error is the estimation of respiratory water loss, which was not directly measured but instead estimated (via Equation 16). Since the total water loss includes both sweat and respiratory losses, any error in estimating the latter affects the calculated water loss and therefore the comparison to the model predictions. However, this impact is limited, as respiratory water loss accounts on average for only ~10% of total water loss in this dataset. Thus, while some uncertainty remains, it is unlikely to influence the overall conclusions. This error could be eliminated in future experiments by directly measuring respiratory water loss, for example, using a breath-by-breath gas analysis system with humidity sensors.

A limitation of the present study is that all three datasets used for model comparison exclusively included male participants. Consequently, the findings may not be fully generalizable to females, as previous research has demonstrated differences in the thermoregulatory sweating responses between sexes [63–69]. Future investigations should also include female populations [70] to increase the robustness and external validity of body-temperature-controlled sweat models. Another limitation of this study is that $\dot{V}O_{2\max}$ data were only available in the HEAT-SHIELD dataset. This restricts the ability to perform a robust residual analysis to assess how the inclusion of this variable affects model predictions.

The highest obtained R^2^ values of the sweat models (even with T_c,set_ or $\overset{ˉ}{T}$_sk,set_ optimization) are approximately 0.5. For higher accuracy, models may require more than only body temperature. Classically, it is understood that afferent signals from deep body core and skin temperature sensors are transmitted and interpreted by (primarily) the hypothalamus, after which efferent signals are sent to the sweat glands to initiate or adjust sweat production [1,22,34,71]. However, research suggests that non-thermal factors can also augment or inhibit neural drive for sweating [71,72]. Review papers from Mekjavic and Eiken [72], and Kenny [69] about the contribution of thermal and non-thermal factors in body temperature regulation, found non-thermal factors such as exercise/postexercise [73,74], dehydration [75], sleep [76], motion sickness [77,78], fever [79], and inert-gas narcosis [80] affect the neural drive for sweating. Other studies found that the light intensity [81–91] and color (e.g. blue versus red) [92,93] also affect the human thermophysiology (and perception) [94]. Among these non-thermal factors, exercise is of particular interest as the dataset consists of experiments performed at various measured exercise intensities (i.e. metabolic rates).

Research shows that the non-thermal neural drive for sweating from exercise mainly arises from three primary mechanisms [71]: 1) central command [95–97], which is a mechanism originating in the brain that provides feedforward autonomic regulation in response to motor activity; 2) muscle mechanoreceptors [97–100], which respond to physical movement and mechanical deformation; and 3) muscle metaboreceptors [101–103], which sense metabolic byproducts (i.e. metabolites) in active muscles. These mechanisms are believed to contribute to the more pronounced underpredictions of WLR by the models in case of high activity levels. It should be noted that non-thermal sweat drive depends on thermal load, and its response is reduced when internal temperatures are elevated [69,104]. Figure 2(a) shows that the HEAT-SHIELD trials, compared to other datasets, show significantly higher activity levels resulting indeed in higher core and skin temperatures (Figures 2(b,c)), a higher water loss rate (WLR, Figure 2(d)), and higher underpredictions (Figures 3 and 4). This suggests that non-thermal factors may also play a significant role in the regulation of sweat production, warranting further consideration in future sweat models. It may also explain the low optimized core temperature setpoint values (36.2°C to 36.6°C). Typically, sweating is not expected to begin at these core temperatures during rest; however, this could result from non-thermal exercise-induced sweat promotion, as the dataset in this study includes only exercising participants and no resting individuals.

**Figure B1.**
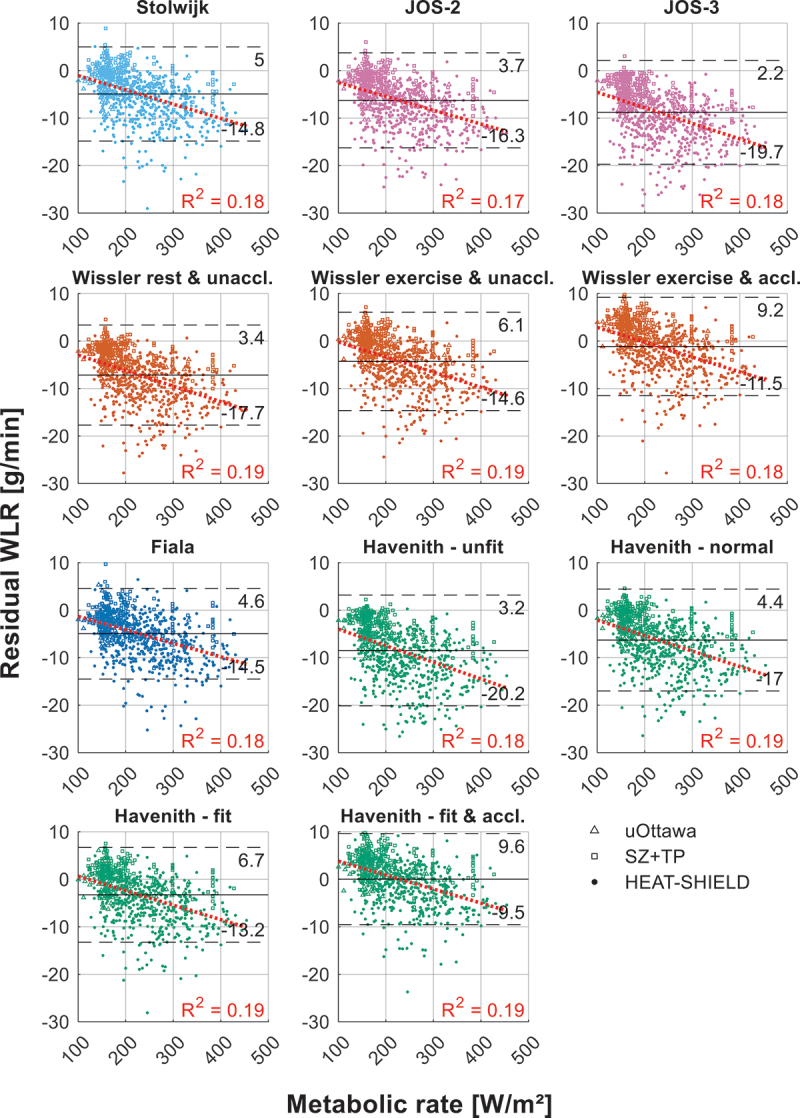
Residual water loss rate (WLR) vs. metabolic rate.

Analysis of the residual WLR and average metabolic rate (Figure B1, Appendix – B) reveals a consistent linear correlation across all models, with all R^2^ values close to 0.2. The negative slopes indicate that underpredictions become larger at higher metabolic rates (and therefore exercise intensities), suggesting that exercise-driven sweat stimulation is not fully accounted for in the models. Since all models exhibit similar correlations, this appears to be a systematic limitation rather than random noise or model-specific errors. However, while an R^2^ value of 0.2 indicates a relatively weak relationship, stronger correlations (i.e. higher R^2^ values) might have been expected if non-thermal exercise-induced sweating played a more dominant role. One possible explanation for the relatively low R^2^ values is the experimental design: the dataset consists of prolonged exercise trials (60–90 min) under stable environmental conditions. In such conditions, thermally induced sweating gradually becomes the dominant driver, reducing the relative contribution of non-thermal exercise sweat drive and, consequently, lowering the observed correlation. Higher R^2^ values might be observed in studies with intermittent or shorter-duration exercise, where non-thermal exercise-driven sweat stimulation likely plays a more prominent role.

The limited predictive accuracy of the models, even after setpoint optimization, likely reflects the inherent complexity of human thermoregulation. A fundamental challenge lies in the fact that we still lack a comprehensive understanding of what the body actually regulates – whether it is core temperature, mean body temperature, or another (integrated) signal such as heat flux [105]. Furthermore, the distribution of temperature sensors across tissues, how the body weights signals from different tissues, and how these inputs are integrated to drive sweating remain poorly understood. Nonthermal factors, such as the rate of temperature change, exercise intensity, hydration status, and even psychological or environmental cues, likely play a significant role in modulating sweat output. This complexity underscores why it is so difficult for models to accurately replicate real-world sweat responses and highlights the need for further research into the physiological mechanisms underlying thermoregulation.

## Conclusion

Multiple sweating formulae predicting sweat rates based on body temperatures were evaluated using an independent dataset. This dataset comprised experiments where participants engaged in exercise (walking or cycling) performed at low to intense exercise intensities, under a range of environmental conditions, and while wearing different types of clothing. Body temperatures and water loss were measured during these trials. Model performance varied significantly, with R^2^ values ranging from −1.3 to 0.5 and RMSE values between 4.9 and 10.4 g/min. While most models performed reasonably well at lower WLRs, they consistently underpredicted higher WLRs, especially models with a higher core temperature setpoint, limiting their accuracy in high-heat-stress scenarios.

Optimization of the models’ core and skin temperature setpoints revealed a preference for a core temperature setpoint between 36.2°C and 36.6°C in all optimized models. However, no consistent setpoint was found for the mean skin temperature, which varied between 28°C and 32°C. The optimized models’ R^2^ values were centered around 0.5, with only modest fluctuations (e.g. 0.43 to 0.54). Accuracy could be further improved by incorporating non-thermal factors such as activity level. The additional neural drive for sweat secretion that arises from various mechanisms induced by exercise could reduce the models’ underpredictions prevalent at higher exercise intensities.

These findings highlight the need for integrating physiological non-thermal factors into future models to enhance predictive accuracy, particularly for applications involving high-intensity physical activity. Future research could aim to separate the effects of activity level (metabolic rate) from core and skin temperature signals, allowing for a more precise understanding of their individual contributions to sweating. In addition, it is recommended to evaluate model differences within a broader physiological and practical context. Specifically, future research should examine models’ effect on the core and skin temperatures, as well as (de)hydration levels. Such analyses could not only highlight the significance of accurate sweat rate predictions but also unveil limitations in existing models. Exploring model calibration using additional individual-specific parameters that influence thermoregulatory responses, such as $\dot{V}O_{2\max}$, acclimation status, and sex, may also help to refine predictive models.

## Abbreviations

bpm: Beats per minute
E_req_: Evaporation required for heat balance
EU: European Union
LoA: Limits of agreement
Q10: Temperature coefficient
R^2^: Coefficient of determination
RMSE: Root mean square error
RSS: Residual sum of squares
SZ: Subzero project
T_c_: Body core temperature
TP: Thermprotect project
$\overset{ˉ}{T}$_sk_
Mean skin temperature
TSS: Total sum of squares
$\dot{V}O_{2\max}$
Maximal oxygen consumption
WBGT: Wet-bulb globe temperature
WBSL: Whole-body sweat loss
WBSR: Whole-body sweat rate
WL: Water Loss
WLR: Water Loss Rate

## Appendix A

Figure A1 shows Bland-Altman plots of the models with optimized setpoint temperatures with T_c_ referring to optimized core temperature setpoint and T_sk_ optimized skin temperature setpoint. The y-axis contains the model’s residual WLR (i.e. predicted minus observed WLR), and the x-axis the mean WLR (i.e. predicted plus observed WLR divided by two). Compared to the original models’ performance (Figure 4), the residuals have decreased, leading to a narrower range in the limits of agreement (LoA). This reduction is expected, as the optimization was performed using the same dataset on which the models are evaluated. More importantly, the linear underprediction trend that is seen in Figure 4 has been significantly reduced in almost all of the optimized models, suggesting that much of the previous error was related to setpoint misalignment rather than an incorrect slope.

## Appendix B

In Figure B1 the residual WLR is plotted versus the metabolic rate of all models. The dotted red line shows the linear least square fit line (linear regression line), with its R^2^ value shown in red, indicating the strength of the correlation. From this figure, it is seen that the residuals of all models are linearly correlated with the metabolic rate and that this correlation is consistent across all models, i.e. negative slope and R^2^≈0.2.
